# Eating Habits and Lifestyles among a Sample of Obese Working Egyptian Women

**DOI:** 10.3889/oamjms.2015.005

**Published:** 2014-12-28

**Authors:** Nayera E. Hassan, Saneya A. Wahba, Sahar A. El-Masry, Enas R. Abd Elhamid, Samia A.W. Boseila, Nihad H. Ahmed, Tarek S. Ibrahim

**Affiliations:** 1 *Biological Anthropology Department, National Research Centre, Dokki (Affiliation ID 60014618), Giza, Egypt*; 2 *Child Health Department, National Research Centre, Dokki (Affiliation ID 60014618), Giza, Egypt*; 3 *Nutrition and Food Science Department, National Research Centre, Dokki (Affiliation ID 60014618), Giza, Egypt*

**Keywords:** Body mass index, obesity, dietary habits, physical activity, sedentary behavior, working women

## Abstract

**BACKGROUND:**

The fundamental cause of obesity and overweight is an energy imbalance between calories consumed and calories expended.

**AIM:**

To figure out food habits and different lifestyle pattern among a sample of Egyptian females working at the National Research Centre.

**METHODS:**

A cross-sectional, descriptive study, including 138 overweight and obese Egyptian females (BMI ≥ 25 Kg/m^2^); working at the National Research Centre; was done. A specific questionnaire was used to gather information regarding lifestyle including dietary habits, physical activity and sedentary behaviour.

**RESULTS:**

The prevalence of overweight among the studied subjects was 27%, while that of obesity was 38%. Missing and or infrequent intake of breakfast at home, frequent consumption of snacks, low serving per day of fruits and vegetables with frequent consumption of sweets, fried food, eating while watching TV and sedentary behaviour were all predictors of obesity and overweight among the current sample.

**CONCLUSION:**

The present study identified several lifestyle factors and improper dietary habits associated with overweight and obesity among Egyptian females. There is a great need to change these habits to avoid the increasing risk of obesity. A national plan of action to overcome obesity is urgently needed to reduce its economic and health burden.

## Introduction

Obesity is becoming a worldwide problem affecting all levels of the society and is thus being globally described as an epidemic. The rapid increase in obesity among the world’s population has become a major public health problem, affecting both developed and developing countries [1].

The obesity epidemic results in a substantial decrease in the quality of life and life expectancy, and it accounts for heavy expenditure in provision of health care. Prevention of childhood obesity has been recognized as a public health priority due to difficulty in the treatment of obesity in adults and the many long-term adverse effects of childhood obesity [2-3]. The development of obesity involves multiple factors, such as improper food consumption, sedentary behaviour, patterns of physical activity, social and environmental variables, and individual susceptibility; determined by unmodified factors such as genetic and biological factors [4].

The rapid increase in world obesity prevalence points to behavioural changes in the 20^th^ century as the main cause. Activities that formerly required high energy expenditure have been replaced by the ease offered by urbanization and industrial and technological progress, leading in turn to lower energy consumption at work, during commuting, and in domestic and leisure activities [5]. Compounding factors in this decreased energy expenditure include globalization of eating habits that favour obesity due to dissemination of refined and processed foods, rich in fat and sugars and served every-growing portions [6].

In many developing countries, the progression of nutritional transition has been detected, characterized by a reduction in the prevalence of nutritional deficiencies and the more expressive occurrence of overweight and obesity not only in the adult population but also among children and adolescents [7].

These characteristics are fundamentally associated with changes in life style and eating habits [8]. Food intake has been associated with obesity not only in terms of the volume of food ingested but also in terms of the composition and quality of diet. Eating habits have changed including low consumption of fruits, green vegetables and milk, increasing consumption of snacks, sweets, and soft drinks; and skipping breakfast resulting in continuous increase in adiposity [9]. Eating habits in addition to environmental differentials represent the most dominant determinant in increasing the tendency of overweight and obesity [10], and a modification in the eating habits may be singleton tactic strategy to a more appropriate weight control [11].

Women in particular have a high prevalence of obesity [12], which negatively impacts their health in many ways. Being overweight or obese increases the relative risk of diabetes and coronary artery disease in women and this is accompanied with a higher risk of low back pain and knee osteoarthritis [13].

So, the purpose of this study was to figure out food habits and different lifestyle patterns among a sample of overweight and obese working females attending the Nutrition Clinic at the National Research Centre.

## Subjects and Methods

### Sample

A cross-sectional study was conducted at the National Research Centre, Giza, during the period from 2012-2013. A total number of 500 females, working at the National Research Centre, were chosen randomly from all categories of the workers to participate in the study after signing a written consent form of the Medical Ethical Committee of NRC. According to their BMI they were classified to normal weight (n= 175, 35%), overweight (n= 135, 27%) and obese (n= 190, 38%), only 138 females continued this study: 59 (42.8 %) were overweight and 79 (57.2%) were obese.

### Methods

Anthropometric measurements including height, weight and Body Mass Index (BMI) were conducted. Trained interviewers collected data on food frequency of different food items and physical activity using a designed food frequency and lifestyle questionnaire specific for this study.

### Anthropometric measurements

Weight was measured using a commercial scale (Seca Scale, Germany) with an accuracy of ± 10 g. The subjects were asked to remove their footwear and wear minimal clothes before weighing them. Standing body height was measured, to the nearest 0.1 cm by using Holtain Stadiometer with the shoulder in a relaxed position and arms hanging freely and without shoes. The scales were recalibrated after each measurement following the recommendations of the International Biological Program [14]. Body Mass Index (BMI) was calculated as body weight in kilograms/height in metere^2^. Females with BMI ≥ 25-29.9 Kg/m^2^ were considered overweight; those with BMI ≥ 30 Kg/m^2^ were considered obese, while those with BMI ≤ 18.5 Kg/m^2^ percentile were considered lean [15].

### Food frequency and lifestyle questionnaire

The forms suggested by Rimm et al [16], after modification to fit the Egyptian setting were used in our study. The forms were prepared and consisted of three different categories: 1- Eating habits, 2- physical activity and 3-sedentary lifestyle. The participants answered the different questions about the frequencies of eating different types of foods that help the purpose of the study (main meals, breakfast, snacks, fruits and vegetables, pickles, fried foods added salts and sweets).

Physical activity as walking, cycling, and different physical activities and sedentary behaviours, as watching TV (eating habits during watching), reading, computer and internet activities were evaluated.

Answers were recorded as closed-ended responses consisting of different categories: rarely, < once/month, once/week, 2-3, 4-5 times/week, or every day.

### Statistical analysis

Data were analyzed using the Statistical Package for Social Sciences (SPSS/Windows Version 16, SPSS Inc., Chicago, IL, USA). Statistical significance was set at P < 0.05. Parametric data were expressed as mean ± SD, while the non-parametric data (qualitative) were expressed as frequency distribution: numbers and percentage of the total. Comparisons between the different non-parametric variables were analyzed using Chi- square test.

## Results

A total number of 138 overweight and obese females with an average age of 21-63 years participated in the current study (59 were overweight and 79 were obese). The mean age for the overweight and obese groups were (38.20 ± 11.48) and (42.17 ± 9.24) respectively. The mean BMI for the overweight group was 27.56 ± 1.4 with a range of 24.9 – 29.30, while that for the obese group was 38.18 ± 6.1 with a range of 29.6 – 63.1 (Table, 1). Comparing the data of overweight and obese group revealed insignificant statistical differences. So, the data was analyzed as one group; overweight and obese; and the results were not different.

**Table 1:** Characteristics of the studied sample (n=138).

Variable	Overweight (N= 59, 42.8 %)	Obese (N= 79, 57.2 %)
Age (years) Mean± SD (Range)	38.2 ± 11.48 (21-59)	42.17 ± 9.24 (25-63)

BMI (kg/m^2^) Mean± SD (Range)	25.56 ±1.43 (24.9- 29.3)	37.18±6.16 (29.9- 36.1)

The different eating habits of the studied women were presented in Table 2. Those who skipped their breakfast meal were 60% of the studied women, while only 39.1% had breakfast, with highly significant differences. Main meal consumption for 3 or more times/week was reported by 94.2 %, snacking was a common habit among the overweight and obese females and its weekly consumption was reported by 67.4% for 2 times or more/week; while 32.6% consumed it only once/week, with highly significant differences. Vegetables and fruits were not frequently consumed, the percentage of subjects who ate them for 1-2 times/week was 44.9% and 52.9%, and those who rarely ate vegetables and fruits were 55.1% and 44.9% respectively with insignificant statistical difference. Fried food was consumed more than 3 times a week by 58.7 % of the population (p = 0.041) and two thirds of the sample (69.9 %) consumed pickles more than 3 times per week, and 87.7% used to add table salt to their food more frequent; with highly significant differences. Sweets were highly consumed, where 79% of the studied subjects were used to eat sweets 3-4 times or even more weekly; with highly significant differences.

**Table 2:** Eating habits of the studied sample.

*Variable*	Frequency of consumption per week	N (n=138)	%	P
*Breakfast*	Rarely	84	60.9	0.011^*^
	More than 1 time	54	39.1	

*Main meal*	Up to 2 times	130	5.8	0.000^**^
	3 times or more	80	94.2	

*Snacks*	Once	45	32.6	0.000^**^
	2 times or more	93	67.4	

*Vegetables*	Rarely	76	55.1	0.233
	1-2 times or more	62	44.9	

*Fruits*	Rarely	65	47.1	0.496
	1-2 times or more	73	52.9	

*Fried Food*	1-2 times	57	41.3	0.041^*^
	3-4 times or more	81	58.7	

*Pickles*	1-2 times	42	30.4	0.000^**^
	3-4 times or more	96	69.6	

*Salts*	1-2 times	17	12.3	0.000^**^
	3-4 times	121	87.7	

*Sweets*	1-2 times	29	21.0	0.000^**^
	3-4 times or more	109	79	

*Having meals with family*	Rarely	5	3.6	0.000^**^
	3-4 times	9	6.5	
	1-2 times	11	8.0	
	Every day	113	81.9	

*Having meals with friends*	Rarely	92	66.7	0.000^**^
	3-4 times	17	12.3	
	1-2 times	5	3.6	
	Every day	24	17.4	

Eating at home with family was a common practice, where 81.9 % shared the main meal with their family every day, 6.5% for 3.4 times/week, 8% for 1-2 times/week, while only 3.6 % of the women rarely joined their families during meals; with highly significant differences. Eating with friends or work colleagues daily was practiced by only 17.4% of the women, 12.3% for 3-4 times/day; 3.6% for 1-2 times/day while 66.7 % hardly shared food with friends (P<0.000).

Table 3 summarizes eating and drinking habits while watching TV. Having meals 3-4 times or more/week while watching TV was performed by 67.41% of the studied sample, while 32.6% rarely have their meals while watching TV; with highly significant differences. Snacks were consumed 1-2 times per week by 58 % of the women, while 42% used to have snacks 3-4 times or more weekly with no statistical difference. Hot drinks were consumed 3-4 times or more/week were tea (63%) followed by coffee (18.2 %) and lastly herbal tea (5.1 %). Soft drinks and fresh juices were rarely consumed while watching TV.

**Table 3:** Eating and drinking habits during watching TV.

Variable	*Frequency/week*	*Total n = 138*		*P*

		*N*	*%*	
Main meal	*1-2 times*	*45*	*32.6*	0.000**
	*3-4 times or more*	*93*	*67.4*	

Snacks	*1-2 times*	*80*	*58.0*	0.061
	*3-4 times or more*	*58*	*42.0*	

Tea	*1-2 times*	*51*	*37.0*	0.002**
	*3-4 times or more*	*87*	*63.0*	

Coffee	*1-2 times*	*113*	*81.9*	0.000**
	*3-4 times or more*	*25*	*18.1*	

Soft drink	*1-2 times*	*109*	*79.0*	0.000**
	*3-4 times or more*	*29*	*21.0*	

Herbal tea and fresh juice	*1-2 times*	*128*	*92.8*	0.000**
	*3-4 times or more*	*10*	*7.2*	

Concerning physical activity and sedentary behaviour (Table 4), the studied sample showed that 37% did not walk at all, 26% walked less than 15 minutes/day while 37% walked more than 15 minutes/day; with significant differences. Performing manual housework activity for more than 2 hours/week was reported by 79.7%, while those who worked less than 2 hours were 20.3%, with highly significant difference; with highly significant differences.

**Table 4:** Physical activity and sedentary behavior of the studied sample.

Variable	Duration	N (138)	%	P
Physical activity			

*Walking time*	never	51	37.0	0.196
	< 15 min/week	36	26.0	
	> 15 min/week	51	37.0	

*Cycling*	Never	137	99.3	0.000**
	up to 60 min/week	1	0.7	

*Housework*	< 2 hrs/week	28	20.3	0.000**
	> 2 hrs/week	110	79.7	

Sedentary behavior				

*TV watching*	< 2hrs/day	91	65.9	0.000**
	> 2 hrs/day	47	34.1	

*Reading*	never	84	60.9	0.000**
	30 min or more/day	54	39.1	

*Computer*	Never	119	86.2	0.000**
	1 hr/day >	5	3.6	
	1 hr/day	14	10.1	

Sedentary behaviours included, reading, using computer and TV watching: 60.9% of the women did not read at all and 39.1% read for only 30 minutes a day; with statistical significant differences. Using the computer for more than 1 hour daily was performed by 10.1%, while 86.2% did not use it at all; with highly significant differences. The percentage of women watching TV for more than 2 hours per day was 34.1%, while those who watched TV for less than 2 hours daily were 65.9%; with highly significant differences.

## Discussion

With the changing food habits and the increasing sedentary lifestyles, the prevalence of obesity has increased markedly in Egypt over the recent decades; with nearly 70 percent of the Egyptian adult population being overweight or obese, Egyptians represent the fattest African populations. [17]. Egypt occupies the 14^th^ place in obesity worldwide, according to the most recent World Health Organization statistics [18]. Egypt Demographic and Health Survey EDHS [19] stated that the proportions classified as obese increased directly with age, from a level of 10 percent among women aged 19 to 65 percent or more among women in the 45-59 age groups. Urban women were more likely to be obese than rural women, and the percentage classified as obese ranged from 25 percent in rural Upper Egypt to 49 percent in the urban Lower Egypt. Women in the highest wealth quintile were almost twice, as likely as, women in the lowest quintile to be obese. The easily available, cheap, high-caloric foods combined with sedentary lifestyles lead to the significant prevalence of overweight and obesity [20].

The aim of this study was to explore the possible lifestyle factors such as eating habits and physical activity leading to overweight and obesity among a sample of working Egyptian females at the National Research Centre. The current data demonstrated that more than one half of the 138 studied subjects were obese (57.2 %), while 42.8 % were overweight.

Skipping breakfast may be related to obesity, as individuals who do not eat early in the morning, may feel hungry later on and then may consume a greater number of calories during the evening hours than individuals who eat consistently throughout the day [21]. The findings of the current study have shown that more than half of present sample (60%) used to skip their breakfast, while 39% have breakfast more than once/week; with highly statistical significant differences. This is similar to the Saudi study, where 68.7% of the women who skipped breakfast were obese, and the rest (31.3%) were non obese (P<0.007) [22]. Current percentage is higher than that reported by Kerkadi [23] in The United Arab Emirates who found that 40.8 % of the respondents did not have breakfast. Twenty percent of the subjects in the study of Cho et al. [24] in The United States skipped their breakfast and an association between skipping breakfast and high BMI was demonstrated.

The results of this study showed that the majority of the sample (94.2%) used to have the main meal more than 3 times per week and 66.7% were used to have their meals at home with their families, contrarily to only 3.6% who used to have their meals outdoors with their friends 1-2 times/week.

The term “snack” refers to all foods and drinks taken outside the context of the three main meals [25]. Although increased snack consumption is often accused for increased prevalence of obesity, snacks can be used to incorporate healthful foods such as fruits and vegetables, being beneficial in dietary weight loss programs [26]. Eating snacks 2 or more times weekly was a common habit in about 67% of current study; the same was detected in the study of Berteus et al [27]. They found that obese subjects were more frequent snackers than reference subjects and women were more frequent snackers than men. A number of cross-sectional studies in the Eastern Mediterranean Region (EMR), on the other hand showed a negative relationship between snacking and obesity [28].

A high percentage (55.1%) of the participants reported that they rarely consumed vegetables; however for fruit consumption more than half of the participants consumed fruits 2-3 times weekly. This finding is in agreement with that of Musaiger and Abuirmeileh [29] who reported a low fruit and vegetable consumption pattern in a random sample of men and women in the seven Emirates of The UAE. Obese women tended to list predominantly carbohydrate/fat sources (doughnuts, cookies, cake) and foods that were sweet.

Seventy nine percent of our study population consumed sweets 3 or more times weekly, which was also reported in many previous studies as those of Amin et al [30] and Yehia et al [2]. Data from self-reported questionnaires revealed that obese individuals had lower odd of consuming fruit/fruit juice/vegetables and had higher odds of consuming sweetened or diet beverages than those of normal weight [31].

In Egypt and other Mediterranean countries, frying food with oil is a traditional cooking procedure. More than 50% of our studied females consumed fried foods; this is in agreement with a study done in Spain, where fried food was positively associated with general and central obesity [32].

Having meals, while watching television, is another contributing factor for obesity. This may lead to overeating, because the type and the amounts of consumed food may be less well self-monitored [2]. In the current study, 67.4% of the females used to have their main meal, 42 % used to have snacks and 63 % used to have tea 3-4 times/week, while watching TV.

Heavy consumption of starchy and fatty foods, without the presence of a health-conscious exercise culture, is a major factor in developing obesity among Egyptian females. Analysis of physical activity pattern has shown that, almost all the participants did not practice any activity. Walking was the only physical activity that the majority of the participants were engaged in. The results of the present study showed that, 37% used to walk more than 15 minutes per day and 37% never used to walk. Housework was considered as a physical activity where, 79% of the participants were engaged in doing manual housework for more than 2 hours per week. According to a large cohort study on women conducted by Hu et al [33] in the States, sedentary behaviors, especially watching TV for prolonged periods was considered as a major risk factor for developing obesity. In the present study, 34.1% of the participants (n= 47) spent more than two-hours the day watching TV.

In summary, the results of this study indicate that the combined prevalence of overweight and obesity among our sample is comparable to that in the developed countries. A high percentage of the overweight and obese samples were engaged in health risk behaviors and unhealthy dietary practices. Further studies should be performed to include Egyptian females of different social, educational and cultural levels.

Obesity and overweigh is becoming an increasing problem presenting a risk to health. Improper dietary habits, such as skipping breakfast, eating during watching TV, low consumption of fruits and vegetables and practicing more sedentary life with absence of any physical activity may be associated with the problem of overweight and obesity among the Egyptian working females.

Implantation of food programs and nutritional health education messages; with incorporation of skills for the proper selection of food; are recommended. This program should focus on how to control obesity using dietary guidelines, physical activity, lifestyle modification and drugs.

## References

[1] World Health Organization. Obesity: preventing and managing the global epidemic. Report of a WHO consultation (2010). World Health organ Tech Rep Ser.

[2] Yahia N, Achkar A, Abdallah A, Rizk S (2008). Eating habits and obesity among Lebanese university students. Nutr J.

[3] Spanos D, Hankey CR (2010). The habitual meal and snacking patterns of university students in two countries and their use of vending machines. J Hum Nutr Diet.

[4] Thompson JL, Allen P, Cunningham-Sabo L, Yazzie DA, Curtis M, Davis SM (2002). Environmental, policy, and cultural factors related to physical activity in sedentary American Indian women. Women Health.

[5] Merchant AT, Dehghan M, Behnke-Cook D, Anand SS (2007). Diet, physical activity, and adiposity in children in poor and rich neighbourhoods: a cross-sectional comparison. Nutr J.

[6] Monteiro CA, Conde WL, Lu B, Popkin BM (2004). Obesity and inequities in health in the developing world. Int J Obes Relat Metab Disord.

[7] O’Dea JA, Wilson R (2006). Socio-cognitive and nutritional factors associated with body mass index in children and adolescents: possibilities for childhood obesity prevention. Health Educ Res.

[8] Wang Y, Monteiro C, Popkin BM (2002). Trends of obesity and underweight in older children and adolescents in the United States, Brazil, China, and Russia. Am J Clin Nutr.

[9] Hanley AJ, Harris SB, Gittelsohn J, Wolever TM, Saksvig B, Zinman B (2000). Overweight among children and adolescents in a Native Canadian community: prevalence and associated factors. Am J Clin Nutr.

[10] Nicklas TA, Baranowski T, Cullen KW, Berenson G (2001). Eating patterns, dietary quality and obesity. J Am Coll Nutr.

[11] Triches RM, Giugliani ER (2005). Obesity, eating habits and nutritional knowledge among school children. Rev Saude Publica.

[12] Sotoudeh G, Khosravi S, Khajehnasiri F, Khalkhali HR (2005). High prevalence of overweight and obesity in women of Islamshahr, Iran. Asia Pac J Clin Nutr.

[13] Kulie T, Slattengren A, Redmer J, Counts H, Eglash A, Schrager S (2011). Obesity and women’s health: an evidence-based review. J Am Board Fam Med.

[14] Tanner JM, Hiernaux J, Jarman S, Weiner J. S, Lourie J. A (1969). “Growth and physique studies,” in Human Biology: A Guide to Field Methods.

[15] WHO (2004). Global strategy on diet, physical activity and health.

[16] Giovannucci E, Colditz G, Stampfer MJ, Rimm EB, Litin L, Sampson L, Willett WC (1992). The assessment of alcohol consumption by a simple self-administered questionnaire. Am J Epidemiol.

[17] Shaheen F, Hathout M, Tawfik A (2004). Prevalence of obesity in Egypt. National survey, final report.

[18] WHO (2008). Action plan for the global strategy for the prevention and control of non-communicable diseases 2008 – 2013.

[19] (2008). Egypt Demographic and Health Survey. Nutritional status. Chapter 14.

[20] Hassan NE, El-Masry SA, Elshebini SM, Al-Tohamy M, Ahmed NH, Abdel Rasheed E, El-Saeed GS, Hassan NM, Zikri EN, El Hussieny MS (2014). Comparison of Three Protocols: Dietary Therapy and Physical Activity, Acupuncture, or Laser Acupuncture in Management of Obese Females. Maced J Med Sci.

[21] Ma Y, Bertone ER, Stanek EJ, Reed GW, Hebert JR, Cohen NL, Merriam PA, Ockene IS (2003). Association between eating patterns and obesity in a free-living US adult population. Am J Epidemiol.

[22] Musaiger A. O, Al-Ahdal E, Musaiger A. O (2010). “Social and dietary factors associated with obesity among women in Saudi Arabia,” in Obesity in the Arab World.

[23] Kerkadi A (2003). Evaluation of nutritional status of United Arab Emirates university female students. Emir J Agric Sci.

[24] Cho S, Dietrich M, Brown CJ, Clark CA, Block G (2003). The effect of breakfast type on total daily energy intake and body mass index: results from the Third National Health and Nutrition Examination Survey (NHANES III). J Am Coll Nutr.

[25] De Graaf C (2006). Effects of snacks on energy intake: an evolutionary perspective. Appetite.

[26] Kong A, Beresford SA, Alfano CM, Foster-Schubert KE, Neuhouser ML, Johnson DB, Duggan C, Wang CY, Xiao L, Bain CE, McTiernan A (2011). Associations between snacking and weight loss and nutrient intake among postmenopausal overweight to obese women in a dietary weight-loss intervention. J Am Diet Assoc.

[27] Bertéus Forslund H, Torgerson JS, Sjöström L, Lindroos AK (2005). Snacking frequency in relation to energy intake and food choices in obese men and women compared to a reference population. Int J Obes (Lond).

[28] Musaiger AO (2011). Overweight and obesity in eastern mediterranean region: prevalence and possible causes. J Obes.

[29] Musaiger AO, Abuirmeileh NM (1968). Food consumption patterns of adults in the United Arab Emirates. J R Soc Promot Health.

[30] Amin TT, Al-Sultan AI, Ali A (2008). Overweight and obesity and their relation to dietary habits and socio-demographic characteristics among male primary school children in Al-Hassa, Kingdom of Saudi Arabia. Eur J Nutr.

[31] Carol E. O’Neil, Priya Deshmukh-Taskar, Jason A. Mendoza, Theresa A. Nicklas, Yan Liu, George Relyea, Gerald S. Berenson (2012). Dietary, Lifestyle, and Health Correlates of Overweight and Obesity in Adults 19 to 39 Years of Age. The Bogalusa Heart Study. Am J Lifestyle Med.

[32] Guallar-Castillón P, Rodríguez-Artalejo F, Fornés NS, Banegas JR, Etxezarreta PA, Ardanaz E, Barricarte A, Chirlaque MD, Iraeta MD, Larrañaga NL, Losada A, Mendez M, Martínez C, Quirós JR, Navarro C, Jakszyn P, Sánchez MJ, Tormo MJ, González CA (2007). Intake of fried foods is associated with obesity in the cohort of Spanish adults from the European Prospective Investigation into Cancer and Nutrition. Am J Clin Nutr.

[33] Hu FB, Li TY, Colditz GA, Willett WC, Manson JE (2003). Television watching and other sedentary behaviors in relation to risk of obesity and type 2 diabetes mellitus in women. JAMA.

